# Olfactory Swab-Derived Human Neuroepithelium Cell Culture as a Novel Model to Study Neurodegeneration-Related Proteins

**DOI:** 10.3390/cells15191824

**Published:** 2026-10-07

**Authors:** Francesco Silvestri, Erika Bronzato, Ilaria Scambi, Valerio Arietti, Luca Sacchetto, Raffaella Mariotti, Gianluigi Zanusso, Matilde Bongianni, Ermanna Turano

**Affiliations:** 1 Department of Neurosciences, Biomedicine and Movement Sciences, University of Verona, 37134 Verona, Italy; francesco.silvestri@univr.it (F.S.); erika.bronzato@univr.it (E.B.); ilaria.scambi@univr.it (I.S.); raffaella.mariotti@univr.it (R.M.); gianluigi.zanusso@univr.it (G.Z.); 2 Department of Surgery, Dentistry, Paediatrics and Gynaecology, Otolaryngology Section, University of Verona, 37134 Verona, Italy; valerio.arietti@univr.it (V.A.); luca.sacchetto@univr.it (L.S.)

**Keywords:** olfactory neuroepithelium, cell cultures, nasal cultures, neurodegeneration

## Abstract

The olfactory neuroepithelium (ON) collected with nasal swabs (NS) is a valuable source for studying brain processes in physiological and pathological conditions, due to its anatomical proximity to the brain and heterogeneous neural cell population. In this proof-of-concept study, we developed a protocol for co-culturing distinct cellular populations from ON, establishing a human olfactory neuroepithelium cell culture (hON) model from healthy individuals (HIs) without differentiation factors. We characterized the cellular composition of ON samples at harvesting, identifying sustentacular cells (Pan-CK^+^), neuronal cells (PGP9.5^+^), and ciliated respiratory cells (βIV-tubulin^+^) by immunofluorescence and flow cytometry. We assessed the expression and distribution of TDP-43, Tau, and α-synuclein, together with their phosphorylated isoforms, to establish a physiological reference profile. Furthermore, Seeding Amplification Assay (SAA) analysis, including disease-positive and healthy control cohorts, confirmed the absence of aggregated protein species in HIs. While p-Tau and p-α-syn localization was preserved between collection and culture, p-TDP-43 displayed neuron-specific nuclear redistribution upon culture, suggesting distinct regulatory dynamics. Longitudinal monitoring revealed that hON cultures maintain a stable neuronal population for up to 21 days, after which cells transition toward a fibroblast-like phenotype. This study establishes NS-derived hON as a feasible model for investigating neurodegenerative mechanisms, providing a baseline for disease comparisons.

## 1. Introduction

The olfactory neuroepithelium (ON) has garnered increasing interest as a source for studying brain processes in both normal and pathological conditions due to the diversity of neural cell types present, which are capable of continuously proliferating and amplifying the population of neural precursors [1].

The ON is a specialized pseudostratified epithelium within the olfactory mucosa (OM), consisting primarily of olfactory sensory neurons (OSNs), sustentacular cells (SCs), and basal cells (BCs). OSNs are the primary cells responsible for odor detection and signal transduction. SCs provide structural and metabolic support to these neurons, maintaining the tissue microenvironment. BCs are located at the base of the neuroepithelium, act as precursor stem cells that can differentiate into either OSNs or supporting cells, thereby contributing to the continuous renewal of the OM [2,3].

OSN axons cross the cribriform plate to reach the olfactory bulb (OB), the first relay station for olfactory information processing. From here, neurons project to several olfactory-related areas, including the anterior olfactory nucleus, piriform cortex, amygdala, and entorhinal cortex. Olfactory information is then further conveyed to associated neocortical regions involved in higher-order processing. Notably, unlike other sensory systems, the olfactory system (OS) thus provides a direct link to the brain [4]. This connection enables the detection of alterations within the OS as early indicators of central nervous system pathologies [5,6]. Early olfactory dysfunction has been shown to correlate with the development of cognitive and motor impairments, particularly in diseases like Alzheimer’s and Parkinson’s [7,8]. Additionally, pathological protein aggregation and deposition have been identified in various regions of the OS, further supporting its role as an early indicator of neurodegenerative processes [9,10]. The human ON cell culture (hON) offers a model for studying neurodegenerative diseases (NDs) from living individuals. In vitro models like induced pluripotent stem cells (iPSCs) have provided novel methods to generate neurons and glial cells from patient cells [11,12]. However, ON cells offer several advantages, including a shorter timeline for obtaining proliferative precursor cells, easier expansion over multiple passages, and lower costs. Unlike iPSCs, ON cells do not require genomic reprogramming, and their neurobiological properties may remain closer to the original tissue [13].

Most morphological studies concerning human OM have traditionally relied on autopsy specimens or invasive biopsies [2,14], both of which are technically complex and carry potential complications. Recently, the use of nasal swabs (NSs) has emerged as a more efficient and non-invasive method for sampling ON. Previous studies from our group have successfully used this method in the diagnosis of prion diseases [14,15,16], as well as for the investigation in olfactory cells of the expression of proteins involved in NDs [9].

The ability to isolate and culture cells derived from ON remains underexplored and challenging. Few studies have provided evidence of the potential and importance of cultivating cells from the ON to investigate specific disorders, particularly in the fields of psychiatric or neurological conditions [17,18]. However, most studies focus on the isolation and separate analysis of single cell types, which reduces the complexity of the in vivo environment by limiting cell–cell interactions. In addition, the use of growth factors and differentiation cocktails, employed to drive stem cell fate or maintain specific cell phenotypes, creates artificial culture conditions.

In this work, we aim to describe a non-invasive method for isolating and establishing a mixed primary culture of distinct cellular populations of the ON by NS derived from healthy individuals (HIs) under minimally supplemented culture conditions, without the use of differentiation factors. The integration of different neural cell lines into a single model aims to preserve in vivo-like cellular interaction, providing a culture system that will be further used to better understand ND pathogenesis.

## 2. Materials and Methods

### 2.1. Participants and Study Design

The study was conducted at the University of Verona and approved by the local Ethics Committee (Prot. n. 221512, 8 April 2025). Written informed consent was obtained from all participants in accordance with the approved protocol.

The study included two distinct cohorts. The primary cohort consisted of 22 healthy individuals (HIs; 13 males and 9 females; mean age 40 ± 3.76 years), enrolled for the establishment and characterization of human olfactory neuroepithelium cell cultures (hON), including immunofluorescence (IF), flow cytometry, and longitudinal culture analyses.

A second independent cohort was included exclusively for Seeding Amplification Assay (SAA) analyses and comprised: 5 healthy individuals (HIs; 4 males and 1 female; mean age 44.4 ± 3.64 years), used as negative controls, 5 patients with Parkinson’s disease (PD; 2 males and 3 females; mean age 64.8 ± 9.68 years), 5 patients with amyotrophic lateral sclerosis (ALS; 3 males and 2 females; mean age 60 ± 7.46 years), and 5 patients with Alzheimer’s disease (AD; 2 males and 3 females; mean age 66 ± 9.28 years) used as positive controls. Samples from this second cohort of subjects were obtained from the University of Verona biobank. Participants were clinically evaluated to exclude neurological or psychiatric disorders, anosmia, recent upper respiratory tract infections, chronic rhinosinusitis, and active allergic rhinitis.

### 2.2. Olfactory Neuroepithelium (ON) Harvesting and Primary Culture Conditions

Nasal swab (NS) sampling was performed by an otolaryngologist under endoscopic guidance using a rigid endoscope. In the primary study cohort, four NS samples were collected from the agger nasi (AN) and middle turbinate (MT) regions of each subject using a FLOQBrush (Copan S.p.A., Brescia, Italy). Three NS samples (2 from AN and 1 from MT) were used for hON culture, whereas the remaining NS sample from AN was used for SAA and immunofluorescence (IF) analyses.

ON cells were exfoliated from swabs by vortexing, and the cellular suspension was centrifuged at 300 g for 5 min. The cellular pellet was resuspended in Dulbecco’s Modified Eagle Medium/Nutrient Mixture F12 (DMEM/F12), supplemented with 20% fetal bovine serum, 1% penicillin/streptomycin, and supplemented with B27 (Thermo Fisher Scientific, Waltham, MA, USA), placed in a 25 cm^2^ flask, and P24 multi-wells (Sarstedt, Nümbrecht, Germany). Cells were grown at 37 °C and 5% CO_2_. After 48 h, the medium was partially refreshed to promote cell adhesion and, in the following days, completely replaced.

Cells were cultured and monitored for morphology and phenotype at multiple time points, starting from the time of harvesting (Th) and throughout a period of approximately 21 days. Observations were performed at around 7 (T1), 14 (T2), and 21 (T3) days (±3 days), according to the inter-individual variability in cell confluence and growth rate.

### 2.3. Pre-Processing of Olfactory Neuroepithelial Cells for SAA Analysis

A total of 42 ON samples were analyzed by SAA for TAR-DNA binding protein 43 (TDP-43), Tau 3R/4R, and α-synuclein (α-syn). Twenty-two samples were obtained from healthy individuals belonging to the primary study cohort. The remaining 20 samples belonged to the independent SAA control cohort and included healthy negative controls (HIs, *n* = 5), patients with Parkinson’s disease (PD, *n* = 5), amyotrophic lateral sclerosis (ALS, *n* = 5), and Alzheimer’s disease (AD, *n* = 5), used as disease-positive controls. Before the analysis, ON samples were thawed, and a disposable inoculating loop (Fisherbrand, Rodano, Italy) was used to transfer ∼2 μL of pellet material (corresponding to ~0.14 μg/μL of total protein) into a tube containing 120 μL phosphate-buffered saline (PBS); the suspension was sonicated at 120 W (Argo Lab, Carpi, Italy) for at least 1 min until the pellet was fully dispersed, and equal volumes of the resulting suspension were added to each reaction well.

### 2.4. Recombinant TDP-43 and α-Syn, Tau (K12CFh) Protein Purification

For TDP-43, the pET-28a plasmid containing the truncated construct (a.a 263–414) of human TDP-43 was briefly expressed in *Escherichia coli* BL21 (DE3) cells (Sigma Aldrich, Milan, Italy). Freshly transformed overnight culture was inoculated into 0.5 L of Luria–Bertani medium with 0.05 mg/mL kanamycin. Protein expression was induced by overnight autoinduction (Sigma-Aldrich, St. Louis, MO, USA). Cells were harvested by centrifugation (3000 g, 30 min, 4 °C) and resuspended in buffer A (6 M Urea, 0.5 M NaCl, 0.05 M imidazole, and 0.02 M Tris-HCl, pH 8.0) by homogenizer (Bandelin Sonopuls, Berlin, Germany). Lysates were centrifuged (8000 g, 60 min, 4 °C) and filtered through 0.22 µm pore filters before loading onto a HiTrap 5 mL column (GE Healthcare, Chicago, IL, USA). The column was washed with Buffer A, followed by step washes with 7 column volumes (CV) of 3% Buffer B (6 M Urea, 0.5 M NaCl imidazole, and 0.02 M Tris-HCl, pH 8.0) and 5 CV of 6% Buffer B. Protein elution was carried out using a linear gradient to 100% Buffer B. Fractions were analyzed via SDS–PAGE analysis and protein stored in 2 M urea, 0.02 M urea and 5% of glycine at −80 °C prior to SAA.

Recombinant α-syn was expressed and purified from the periplasmic fraction as reported [19]. Human K12CFh recombinant tau substrate was purified as described by Metrick et al. [20].

### 2.5. Seeding Amplification Assays (SAAs) in ON

ON samples were tested for TDP-43, α-Syn, and Tau SAA using previously published protocols [19,20,21] with minor modifications as detailed below. For TDP-43 and α-Syn SAA, each well was seeded with 2 μL of ON homogenate at the specified dilution into black 96-well plates with a clear bottom (NUNC, ThermoFisher, Waltham, MA, USA), preloaded with six silica beads (0.8 mm, molecular biology grade; OPS Diagnostics); for Tau SAA, 1 μL ON samples were seeded into black 384-well plates (NUNC, ThermoFisher, Waltam, MA, USA). Plates were sealed and incubated in a BMG/Omega Fluostar plate reader (BMG, Ortenberg, Germany) under cycles of shaking and rest, with bottom-read ThT fluorescence (450 ± 10 nm excitation, 480 ± 10 nm emission) measured at regular intervals throughout the observation period. Assay-specific conditions, including substrate concentrations, are summarized below.

For TDP-43 SAA, the reaction mix contained 0.5 M guanidine hydrochloride (Gdn-HCl), 25 mM Tris-HCl, 5 mM EDTA, and recombinant TDP-43 at 0.005 mg/mL (0.34 μM). A volume of 98 μL of reaction mix was added to each well for a final reaction volume of 100 μL. Plates were incubated at 40 °C for 30 h with cycles of 15 s shaking (100 rpm, double orbital) and 30 min rest, with fluorescence readings taken every 30 min [21].

For α-Syn SAA, the reaction buffer consisted of 40 mM phosphate buffer (pH 8), 170 mM NaCl, 0.0015% SDS, and 0.1 mg/mL recombinant α-Syn (in-house). A volume of 98 μL of buffer was added to each well for a final reaction volume of 100 μL. Plates were incubated at 42 °C for 30 h with cycles of 1 min shaking (400 rpm, double orbital) and 1 min rest, with fluorescence readings taken every 45 min [19].

For K12 Tau-SAA, the reaction buffer consisted of 40 mM HEPES (pH 7.4), 400 mM NaF, 40 μM heparin, and 0.1 mg/mL K12 CFh substrate (6.5 μM). A volume of 49 μL of buffer was added to each well for a final reaction volume of 50 μL. Plates were incubated at 42 °C with cycles of 1 min shaking (500 rpm, orbital) and 1 min rest, with fluorescence readings taken every 15 min for 30 h [20].

### 2.6. Positive and Negative SAA Criteria

Single reactions were considered positive when their fluorescence exceeded a threshold defined as the mean fluorescence of the negative controls plus 4 standard deviations (SD), calculated for each plate. This approach follows the criteria described in previous SAA studies [22,23,24], using a more stringent multiplier (>3 SD) to maximize specificity [25]. ON samples were considered positive when at least two out of three replicate wells exceeded the threshold within the 30-h cut-off. Samples were considered negative when none of the replicate wells exceeded the threshold. Samples that tested positive in only 1 of 3 replicate wells were considered inconclusive and retested. A 30-h cut-off was empirically defined based on spontaneous substrate-only fluorescence increases beyond this time, and each run included substrate-only controls and previously characterized positive and negative samples.

### 2.7. Immunophenotypic Characterization of ON by Immunofluorescence

The twenty-two subjects from the primary healthy cohort were analyzed for immunophenotypic characterization at Th. A part of the cellular pellet used for SAA analysis was fixed with Diacyte (Diapath S.p.A., Martinengo, Italy) for at least 2 h at 4 °C and then washed twice with Phosphate-buffered saline (PBS). Cells were counted using Countess II FL Automated Cell Counters (Thermo Fisher Scientific, Waltham, MA, USA), then the suspension was centrifuged onto slides using a cytospin (CYTOSPIN IV, AHSI, Bermareggio, Italy) and kept frozen until IF was carried out. For immunostaining, cells were permeabilized, and non-specific sites were blocked with a blocking solution (0.3% TRITON X-100 and 5% Normal Goat Serum or Normal Donkey Serum) for 1 h at room temperature (RT). Primary antibodies (Table 1) were diluted in blocking solution and incubated overnight at 4 °C. Appropriate Alexa Fluor-conjugated secondary antibodies (Thermo Fisher Scientific, Waltham, MA, USA) were used for 1 h at RT. Nuclei were stained with DAPI (Sigma-Aldrich, St. Loiuis, MO, USA). For each HI, 2–3 slides were stained, and images were acquired using a Leica THUNDER Imager Tissue microscope, Leica Microsystem (Wetzlar, Germany).

For the immunophenotypic characterization of primary cultures, cells were grown on coverslips pre-treated with Geltrex (Thermo Fisher Scientific, Waltham, MA, USA) and fixed with a 4% paraformaldehyde (PFA) solution at different time points. Following fixation, the subsequent IF steps were performed as described above for the staining protocol of harvested cells. In addition to the antibodies specified in Table 1, phalloidin conjugated to Alexa Fluor™ 488 (Invitrogen, Carlsbad, CA, USA; Cat# A12379) was used as a marker of filamentous actin (F-actin). IF analyses were performed to assess protein localization and distribution rather than to quantitatively compare the expression levels of total and phosphorylated protein species.

### 2.8. Flow Cytometry (FC) Analysis

Cell yield and overall viability of freshly harvested single-cell suspensions were quantified post-harvesting by FC. Cell suspensions were stained with Hoechst 33342 (Thermo Fisher Scientific, Waltham, MA, USA) to identify total nucleated events and 7-Aminoactinomycin D (7-AAD; Thermo Fisher Scientific, Waltham, MA, USA) to discriminate membrane-compromised dead cells. Samples were loaded onto multi-well plates and acquired with LSRFortessa X-20 flow cytometer (BD Bioscience, San Jose, CA, USA) using a fixed acquisition volume of 100 μL. Gated single-cell events were analyzed to exclude doublets and cell debris, allowing the calculation of absolute cell concentrations (cells/100 µL). To characterize the immunophenotype of ON cells, a subset of five HIs (3 male, 2 female) were pelleted at 300 g for 5 min and then stained with the *LIVE*/*DEAD* Fixable Near IR viability kit (Thermo Fisher Scientific, Waltham, MA, USA) according to the manufacturer’s instructions. Cells were fixed and permeabilized using the FIX/PERM kit (BD Bioscience, San Jose, CA, USA). Subsequently, cells were centrifuged at 400 g for 5 min and incubated with anti-PGP9.5, βIV-tubulin, βIII-tubulin, Pan-CK, and Vimentin primary antibodies diluted in 1% bovine serum albumin (BSA) for 1 h at RT. Then, the cells were washed with 1% BSA and incubated with the appropriate Alexa Fluor-conjugated secondary antibodies (Thermo Fisher Scientific, Waltham, MA, USA) (Table 1). Cells were finally resuspended in 1% BSA for antigen expression analysis. To exclude cell debris, nuclei were stained with 0.5 mg/mL DAPI immediately before cell acquisition. A FC gating strategy was used to define cell populations of interest. Specifically, live cells were selected as singlets, DAPI^+^, and *LIVE*/*DEAD^−^*. Cell cycle distribution was further assessed by propidium iodide (PI) staining as follows. Briefly, 2.5 × 10^5^ cells were harvested by centrifugation, and the resulting pellet was fixed in ice-cold 70% ethanol, followed by incubation for 30 min at 4 °C on a rotating mixer. Cells were subsequently washed with 1× PBS and centrifuged at 1590 g for 20 min at room temperature. The pellet was resuspended in staining solution containing 1% PI and 0.8 mg/mL RNase A, and incubated for 1 h at 37 °C with shaking prior to FC analyses.

### 2.9. Statistics and Data Analysis

Pan-cytokeratin (Pan-CK), Protein gene product 9.5 (PGP9.5), βIV-tubulin, βIII-tubulin, and vimentin median fluorescence intensity (MFI) levels were obtained by subtracting the corresponding fluorescence from one control (FMO, secondary antibody only). Furthermore, the resulting percentage of positive cells was applied to the total number of cells loaded per tube, thereby normalizing for differences in sample loading input across experiments. Samples were acquired using an LSRFortessa X-20 (BD Bioscience, San Jose, CA, USA), while data were analyzed with FlowJo software (v10.1) (BD Bioscience, San Jose, CA, USA). The experiment was performed using independent biological replicates (*n* = 5). MFI values and percentage of positive cells were analyzed for each marker separately using a repeated-measures one-way ANOVA followed by Tukey’s multiple-comparison test. Vimentin data were analyzed using a one-way ANOVA, followed by Tukey’s multiple-comparison test. βIII-tubulin, PGP9.5, and cell proliferation data were compared using a paired two-tailed Student’s *t*-test. Unless otherwise specified, all data are expressed as mean ± standard error of the mean (SEM). All statistical analyses were performed using GraphPad Prism Inc. (v8) (San Diego, CA, USA) Differences were considered statistically significant at *p* < 0.05.

## 3. Results

### 3.1. Cellular Composition and Phenotypic Characterization of Nasal Swab Samples at the Time of Harvesting (Th)

Olfactory neuroepithelial cells (ONs) were successfully isolated from nasal swabs (NS). Quantifications performed on individual swabs (from the four collected per subject) yielded a mean of 3.17 × 10^5^ ± 5.1 × 10^4^ total cells, with an average post-harvesting viability of 17.2% ± 2.6% as determined by vital staining. Following this initial harvesting and yield assessment, we proceeded with the phenotypic characterization of the isolated populations.

**Figure 1:**
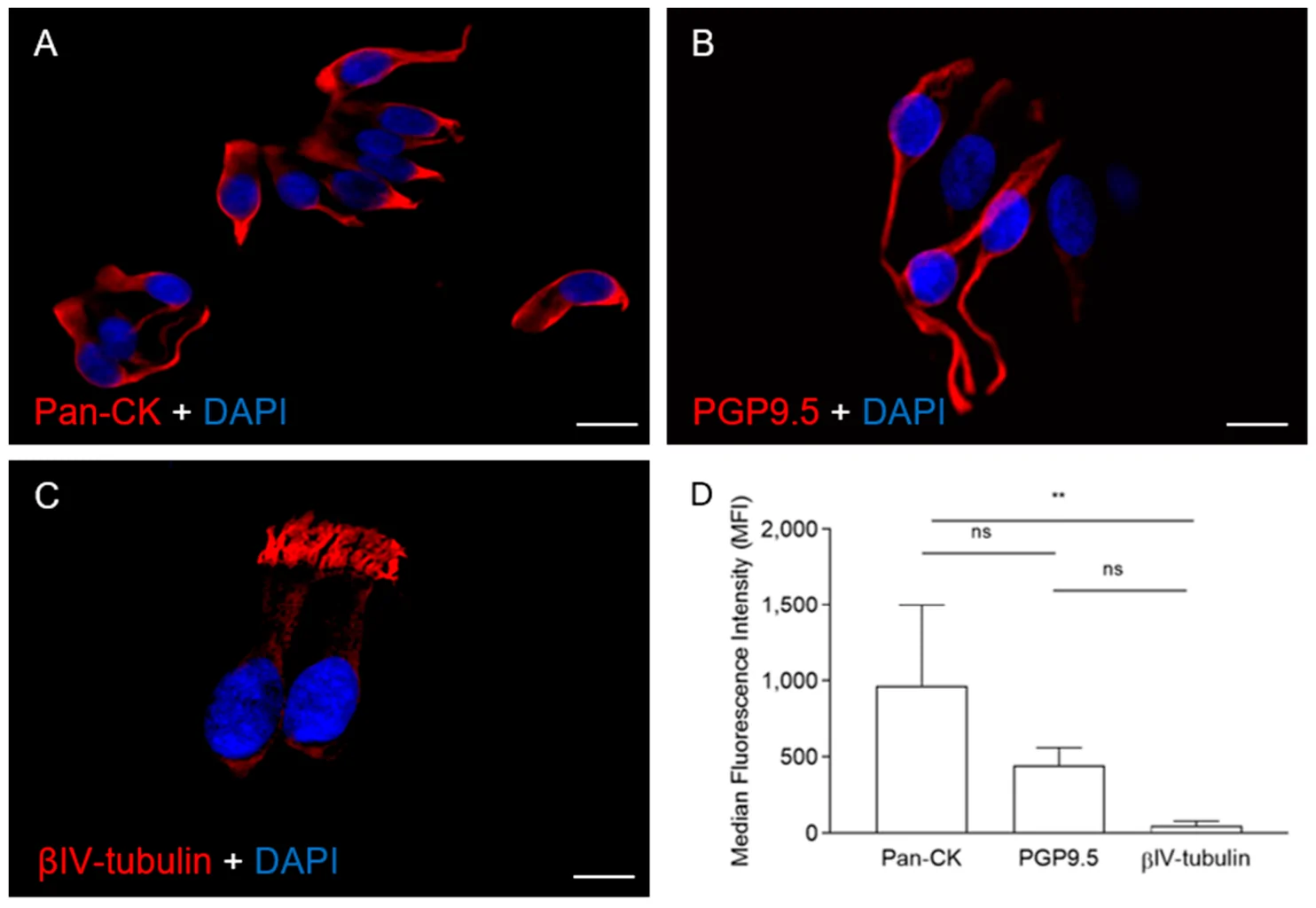
Representative immunofluorescence images showing (**A**) Pan-CK expression in sustentacular cells, (**B**) PGP9.5 expression in neuronal cells, and (**C**) βIV-tubulin expression in respiratory cells. Nuclei were counterstained with DAPI. Scale bar = 10 μm. Images are from one healthy individual out of 22 analyzed; 2–3 slides were stained per individual. The same staining pattern was observed across slides and subjects. (**D**) Median fluorescence intensity (MFI) of Pan-CK, PGP9.5, and βIV-tubulin markers of cells at time of harvesting obtained by flow cytometry. Analysis was performed using a one-way ANOVA. Data are shown as mean ± SEM of five healthy individuals (*n* = 5) (** *p* < 0.01). ns: not significant.

We assessed the cellular characterization of ON cells immediately after NS by immunofluorescence (IF) to identify the cellular population at the Th. The analysis revealed a heterogeneous cell population composed of cell types of different origins. The use of pan-cytokeratin (Pan-CK) antibody revealed positivity in the cytoplasm of non-neural cells, indicating the presence of sustentacular cells (SCs). These cells exhibited a diffuse cytoplasmic positivity and an elongated columnar morphology (Figure 1A). To further characterize this Pan-CK^+^ population, additional markers were assessed, including CK8 and CK18, typically expressed in low-columnar/cuboidal and terminally differentiated epithelial cells, respectively, and CK5, consistent with a basal cell phenotype. These findings confirmed the presence of sustentacular, differentiating, and basal epithelial cells within the ON (Supplementary Figure S1). Furthermore, the presence of olfactory sensory neurons (OSNs) was assessed using the Protein gene product 9.5 (PGP9.5) antibody, exhibiting a typical morphology with an oval soma and cytoplasmic staining highlighting the cell bodies and axonal processes (Figure 1B). The PGP9.5 antibody provided a more specific signal compared to commonly used neuronal markers such as βIII-tubulin (Supplementary Figure S2). The expression of βIV-tubulin in the NS samples was also observed in ciliated positive cells typical of respiratory epithelium (Figure 1C). Moreover, we performed flow cytometry to distinguish the phenotypes of cellular populations present at the time of harvesting (Th). We detected a high presence of Pan-CK^+^ cells at Th (60.4 ± 5.5%), and a neuronal component was also detected (31.2 ± 4.2%), confirming the immunophenotypic characterization. Additionally, a low proportion of βIV-tubulin^+^ cells was observed (5.2 ± 2.6%), confirming the correct anatomical area of sampling. MFI values for all markers are reported in Figure 1D, where βIV-tubulin showed significantly lower intensity compared to Pan-CK (*p* = 0.0027).

### 3.2. Distribution of Neurodegeneration-Related Proteins in the Olfactory Neuroepithelium of HIs at the Time of Harvesting

To further characterize the ON cells identified in NS samples from healthy individuals (HIs), immunostaining was performed for TDP-43, α-syn, and Tau, along with their respective phosphorylated forms (pTDP-43, p-α-syn, and p-Tau). Total and phospho-specific antibodies showed distinct localization patterns, validating their high specificity.

**Figure 2:**
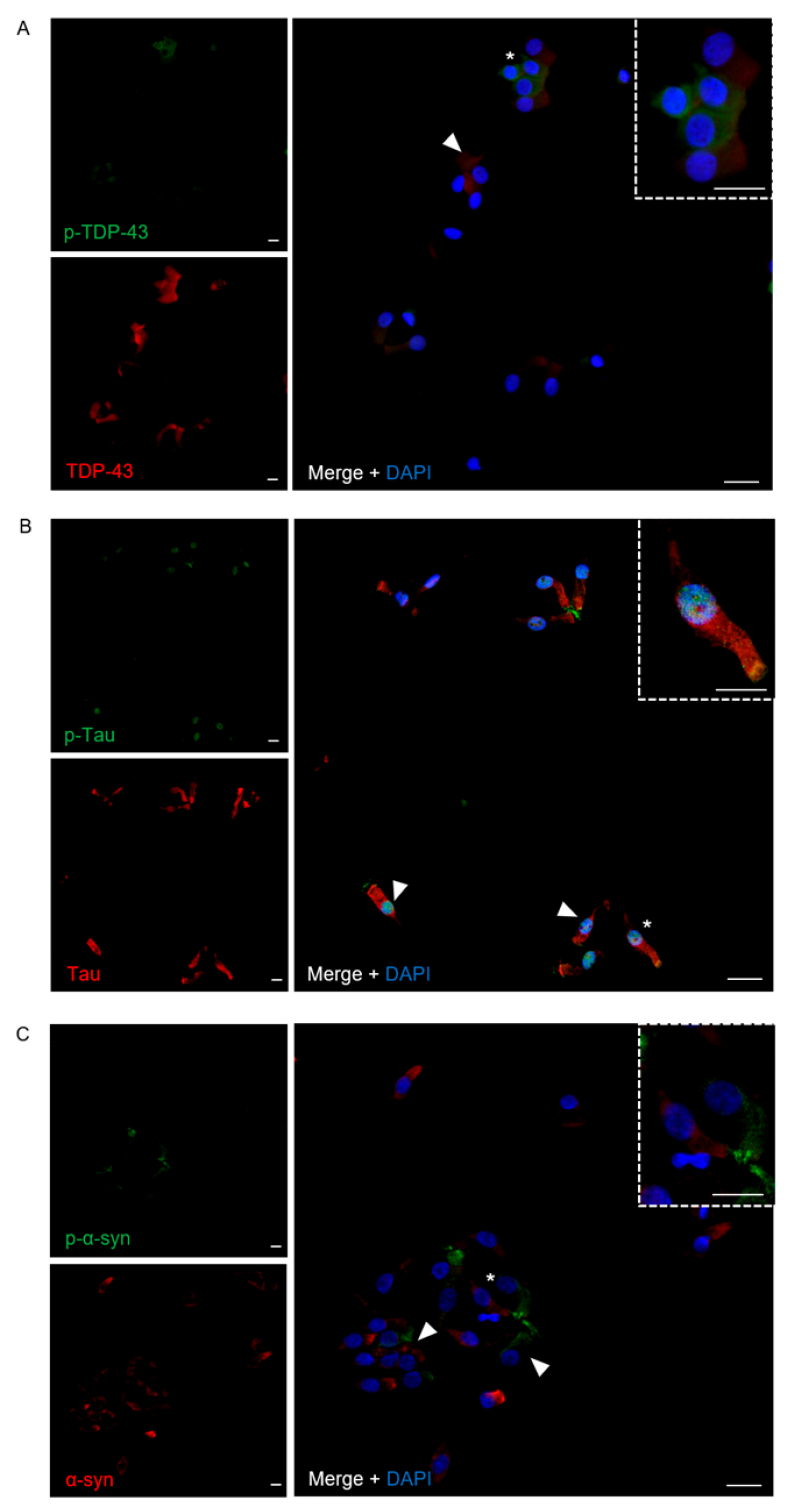
Representative immunofluorescence images of olfactory epithelium cells at the time of harvesting, showing staining of (**A**) pTDP-43 and TDP-43, (**B**) p-Tau and Tau, and (**C**) p-α-syn and α-syn. Arrows indicate positive staining. Insets show magnified views of the indicated regions (*). Nuclei were counterstained with DAPI (blue). Scale bars = 20 µm and 5 µm (inset). Images are from one healthy individual out of 22 analyzed; 2–3 slides were stained per individual. The same staining pattern was observed across slides and subjects.

TDP-43 and pTDP-43 immunoreactivity were localized in the cytoplasm, whereas Tau and p-Tau signals were confined to the cytoplasm and nucleus, respectively (Figure 2A,B). Immunostaining for α-syn and p-α-syn revealed a predominantly cytoplasmic distribution, with a faint signal also detected in the apical region of the cells (Figure 2C).

**Figure 3:**
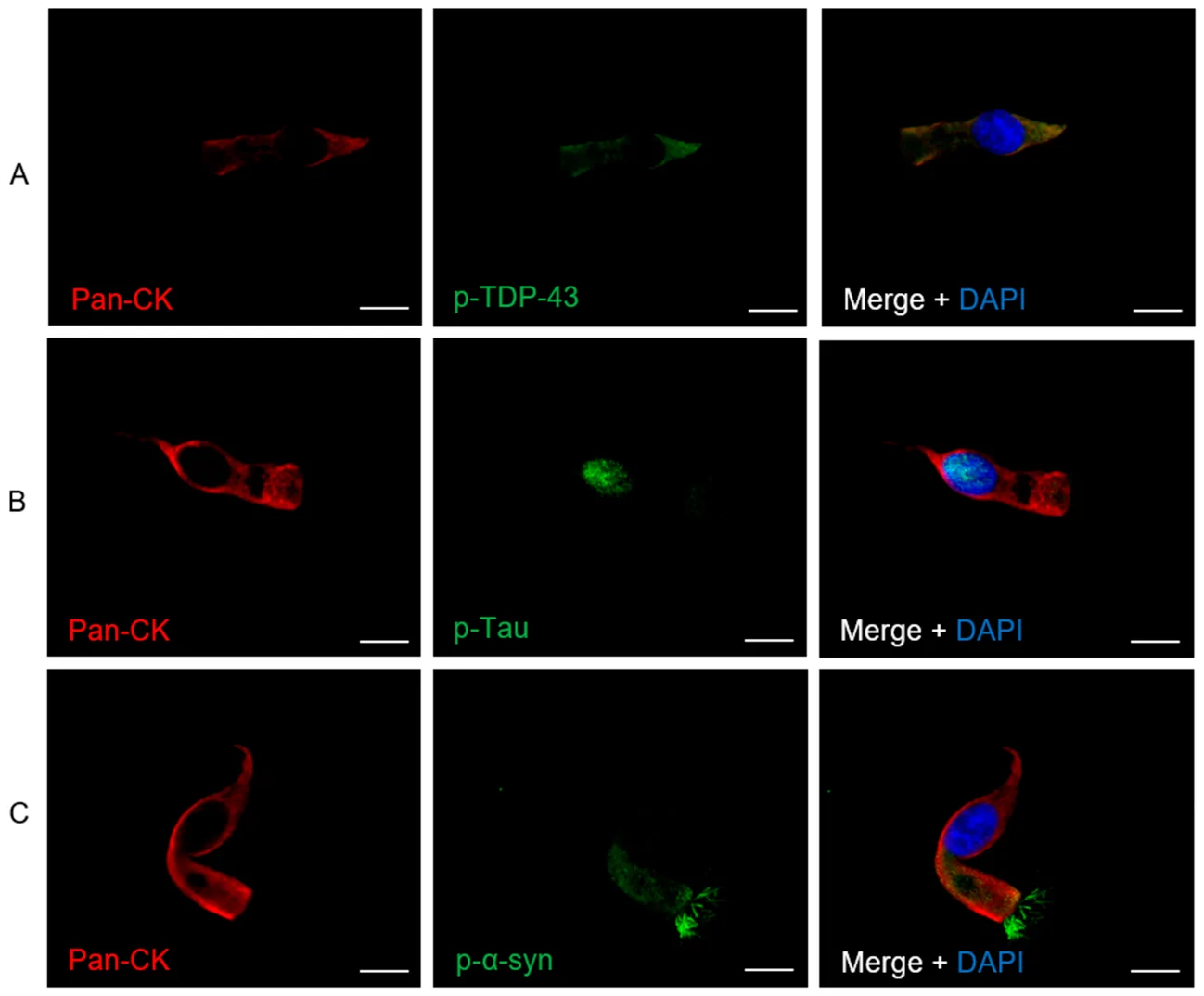
Representative immunofluorescence staining in sustentacular cells Pan-CK^+^ at time of harvesting (Th) of cells from healthy individuals for (**A**) p-TDP-43, (**B**) p-Tau, and (**C**) p-α-syn (Scale bar = 10 μm). Nuclei were counterstained with DAPI. Images are from one healthy individual out of 22 analyzed; 2–3 slides were stained per individual. The same staining pattern was observed across slides and subjects.

**Figure 4:**
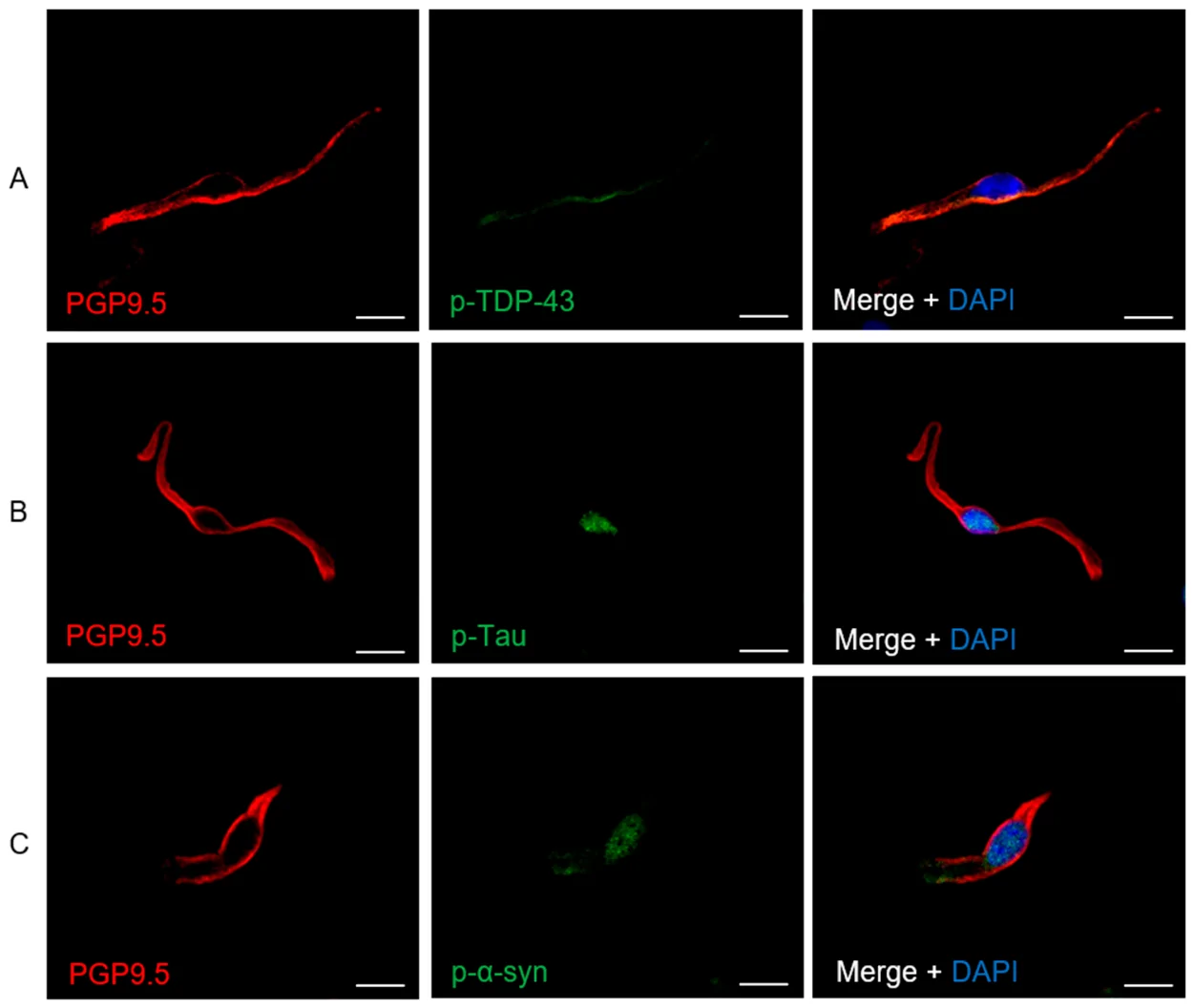
Representative immunofluorescence staining in neuronal cells PGP9.5^+^ at the time of harvesting (Th) of cells from healthy individuals for (**A**) p-TDP-43, (**B**) p-Tau, and (**C**) p-α-syn (Scale bar = 10 μm). Nuclei were counterstained with DAPI. Images are from one healthy individual out of 22 analyzed; 2–3 slides were stained per individual. The same staining pattern was observed across slides and subjects.

Since phosphorylated isoforms of TDP-43, Tau, and α-syn are considered pathological hallmarks of neurodegeneration, we assessed the subcellular distribution of these modified proteins in both sustentacular and neuronal cell populations. In sustentacular Pan-CK^+^ cells, p-TDP-43 was weakly detected in the cytoplasm (Figure 3A), p-Tau in the nucleus (Figure 3B), and p-α-syn was faintly detected in the cytoplasm and displayed slightly stronger labeling in the cilia (Figure 3C). In PGP9.5^+^ neurons, faint cytoplasmic p-TDP-43 immunoreactivity was detected, whereas p-Tau and p-α-syn were localized in the nucleus (Figure 4A,B). A weak cytoplasmic signal was also observed for p-α-syn (Figure 4C).

### 3.3. Seeding Amplification Assay (SAA) Confirms Absence of Fibrillary Protein Forms in Healthy Individuals

**Figure 5:**
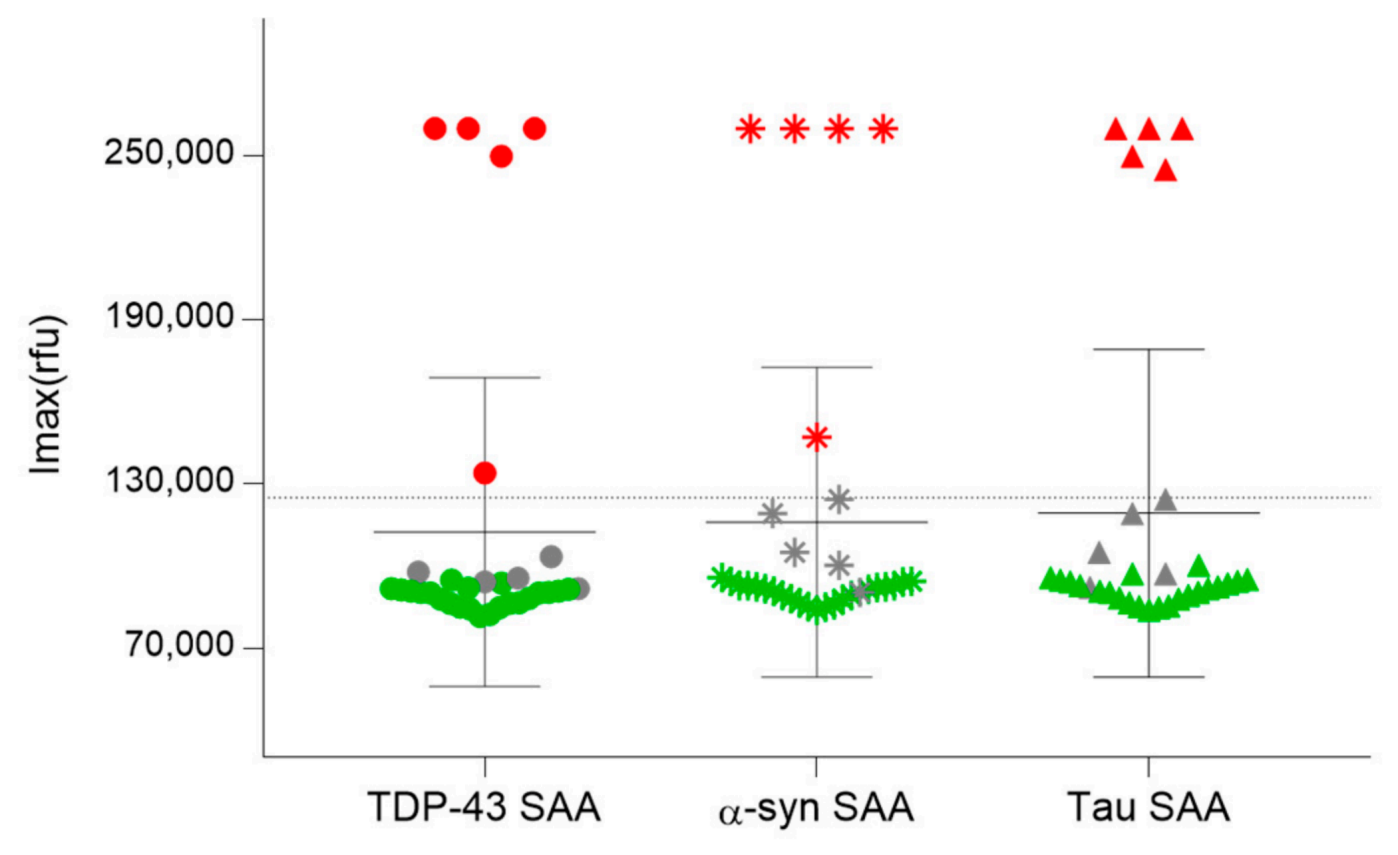
Average maximum fluorescence values in each column (Imax, rfu) are shown for HIs (green, *n* = 22), negative controls (grey, *n* = 5), and *n* = 15 positive controls from patients with neurological disease (red, *n* = 5 ALS, *n* = 5 PD, and *n* = 5 AD). The dashed line marks the fluorescence threshold for positive results. Bars represent the average ± standard deviation for each case.

All ON samples collected from healthy individuals (HIs) at Th and their relative negative and positive controls were tested by SAA for TDP-43, α-syn and Tau to confirm the absence of fibrillary protein forms associated with neurodegenerative disease. Notably, no increase in maximum fluorescence value (Imax) was observed in ON samples from either the HIs cohort (*n* = 22) or the negative control cohort (*n* = 5), indicating the absence of protein aggregates in these samples. In contrast, an increase in Imax value above the set threshold was reached within 30 h in ON samples from patients with neurological disease (*n* = 15) (*n* = 5 ALS, *n* = 5 PD, and *n* = 5 AD) (Figure 5).

### 3.4. Olfactory Neuroepithelium Cells from HIs Can Be Maintained in Culture

**Figure 6:**
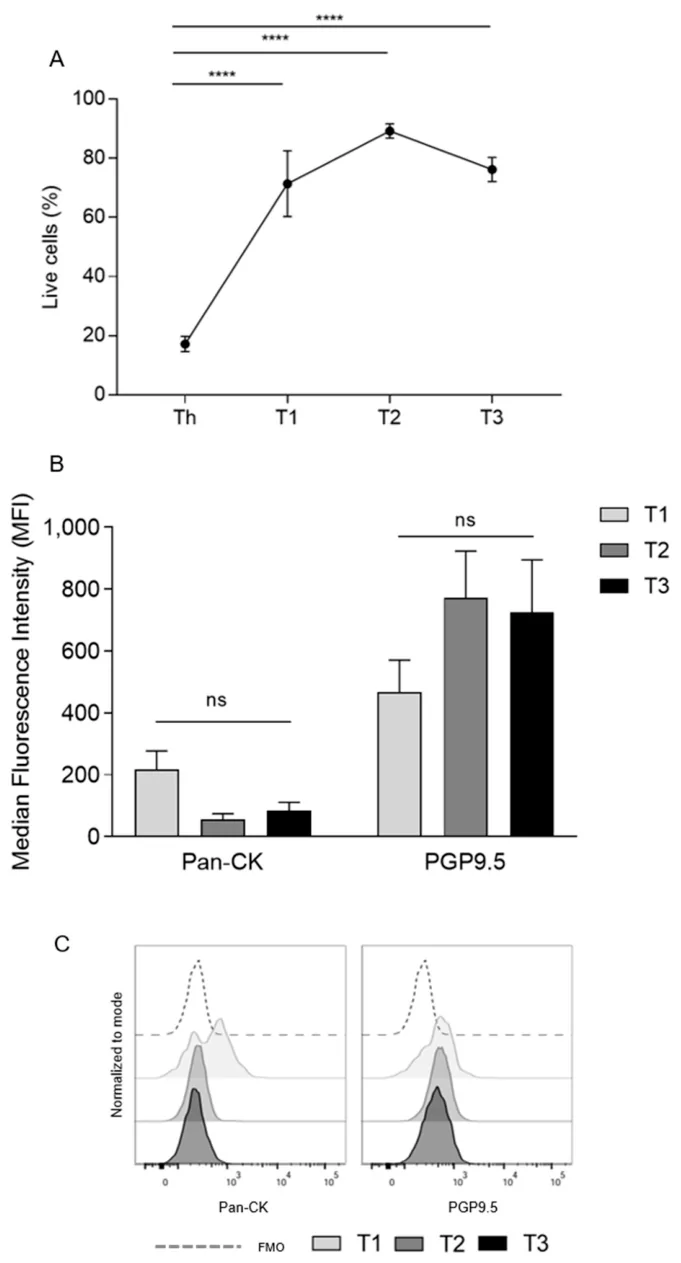
(**A**) Vitality of cells collected with NS at the time of harvesting and in culture of healthy individuals. Data shown mean ± SEM. Analysis was performed using one-way ANOVA (**** *p* < 0.001). (**B**) Median fluorescence intensity (MFI) of Pan-CK, PGP9.5 marker of hON at different time points. Analysis was performed using a one-way ANOVA. Data are shown as mean ± SEM of healthy individuals (*n* = 5). (**C**) Histograms of the normalized fluorescence intensities of the described markers from a representative healthy individual out of five analyzed. ns: not significant.

After NS collection from HIs, the samples were placed in culture, and different cell populations displaying heterogeneous phenotypes and morphologies were observed by brightfield microscopy. A large number of cells initially exhibited a round or oval morphology with the abundance of erythrocytes, which were lysed and disappeared from the culture in the following days. After 3 days, the cells adhered to the plastic surface and started to proliferate, gradually adopting a more elongated or fusiform shape (Supplementary Figure S3). The culture was monitored up to around 21 days (T3). The percentage of live cells at the time of harvesting was 17.2%, with a significant increase at T1 (71.4%) and remaining constant until T3 (*p* < 0.0001) (Figure 6A).

We performed flow cytometry to assess the maintenance of both SCs and OSNs in culture and their dynamic changes over time. Pan-CK expression significantly decreased over time in terms of MFI (Th vs. T2 *p* = 0.0049 and Th vs. T3 *p* = 0.00497), accompanied by a parallel reduction in the percentage of Pan-CK^+^ cells (Th vs. T2 *p* = 0.013 and Th vs. T3 *p* = 0.001). In contrast, the neuronal population identified by PGP9.5 remained stable throughout the culture period, in terms of both MFI and percentage of positive cells (Figure 6 and Table 2).

To visualize the overall cellular morphology independently of PGP9.5-specific signal, phalloidin (F-actin) co-staining was performed, confirming the full extent of the neurite and revealing a growth-cone-like widening at its distal tip (Supplementary Figure S4). The maturation stage of the neuronal population at T1 was further characterized by double staining for GAP-43 (a marker of immature/growing neurons) and PGP9.5, as well as OMP (a marker of mature olfactory neurons) and GAP-43, revealing the coexistence of neurons at different maturation stages within the culture (Supplementary Figure S5).

### 3.5. Cell Cycle Dynamics of Primary hON Cells at Harvesting and in Culture

**Figure 7:**
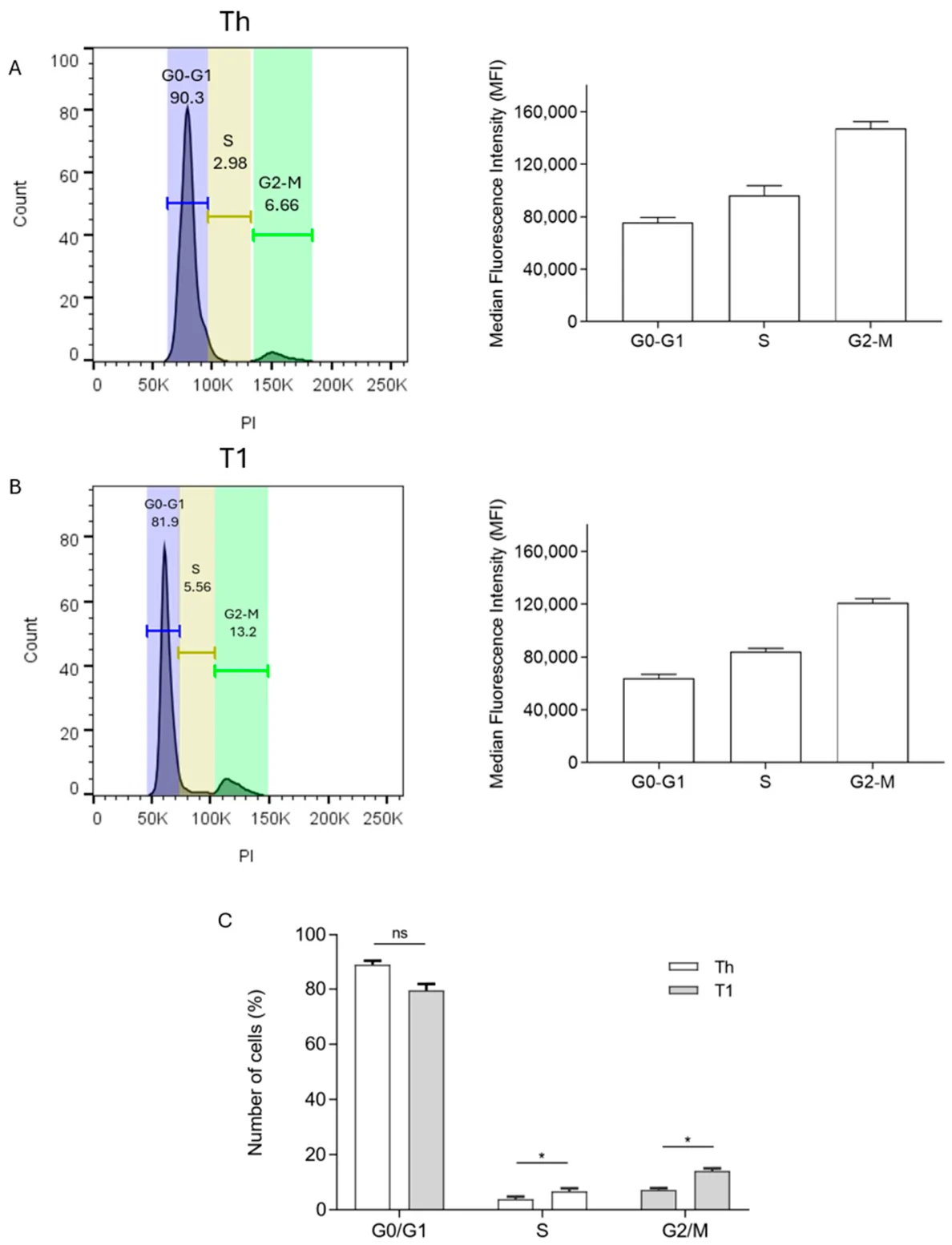
Representative propidium iodide (PI) DNA staining flow cytometry profiles of olfactory neuroepithelium (ON) cells from one healthy individual, out of the three analyzed, immediately after harvesting (Th) (**A**) and after initial culture stabilization (T1) (**B**). Gates highlight the percentage of cells in the G0/G1 (blue), S (yellow), and G2/M (green) phases. The histograms on the right show the median fluorescence intensity (MFI) of PI in each cell cycle phase at Th and T1, expressed as mean ± SEM of three healthy individuals (*n* = 3). (**C**) The histogram compares the percentage of cells in the G0/G1, S, and G2/M phases at Th and T1, expressed as mean ± SEM (*n* = 3). ns, not significant; * *p* < 0.05 (Th vs. T1, paired two-tailed Student’s *t*-test).

Because the transition from harvesting (Th) to initial culture (T1) corresponds to the critical window of cellular viability recovery, we examined whether this process was accompanied by active cell cycle progression using propidium iodide (PI) flow cytometry. Quantitative gating and MFI analysis of PI DNA staining confirmed accurate cell cycle discrimination. At Th, the population was mostly quiescent, with 88.9% of cells in G0/G1, 3.8% in S-phase, and 7.2% in G2/M exhibiting the expected two-fold increase in DNA content relative to G0/G1 (Figure 7A). At T1, the G0/G1 fraction decreased to 79.1%, while cycling populations expanded: S-phase doubled to 6.7%, and G2/M doubled to 14.1% (Figure 7B).

Quantitative analysis of cell cycle distribution confirmed a significant transition into active proliferation upon in vitro culture. Specifically, the percentage of cells in both S phase and G2/M phase increased significantly from Th to T1 (*p* = 0.04 and *p* = 0.02, respectively), accompanied by a decrease in the quiescent G0/G1 population (Figure 7C).

### 3.6. Distribution of Neurodegeneration-Related Proteins in the hON Culture

We further examined the expression and localization of neurodegeneration-associated proteins in both Pan-CK^+^ and PGP9.5^+^ cells at approximately 7–10 days in culture (T1), at which both populations coexisted in substantial proportions.

**Figure 8:**
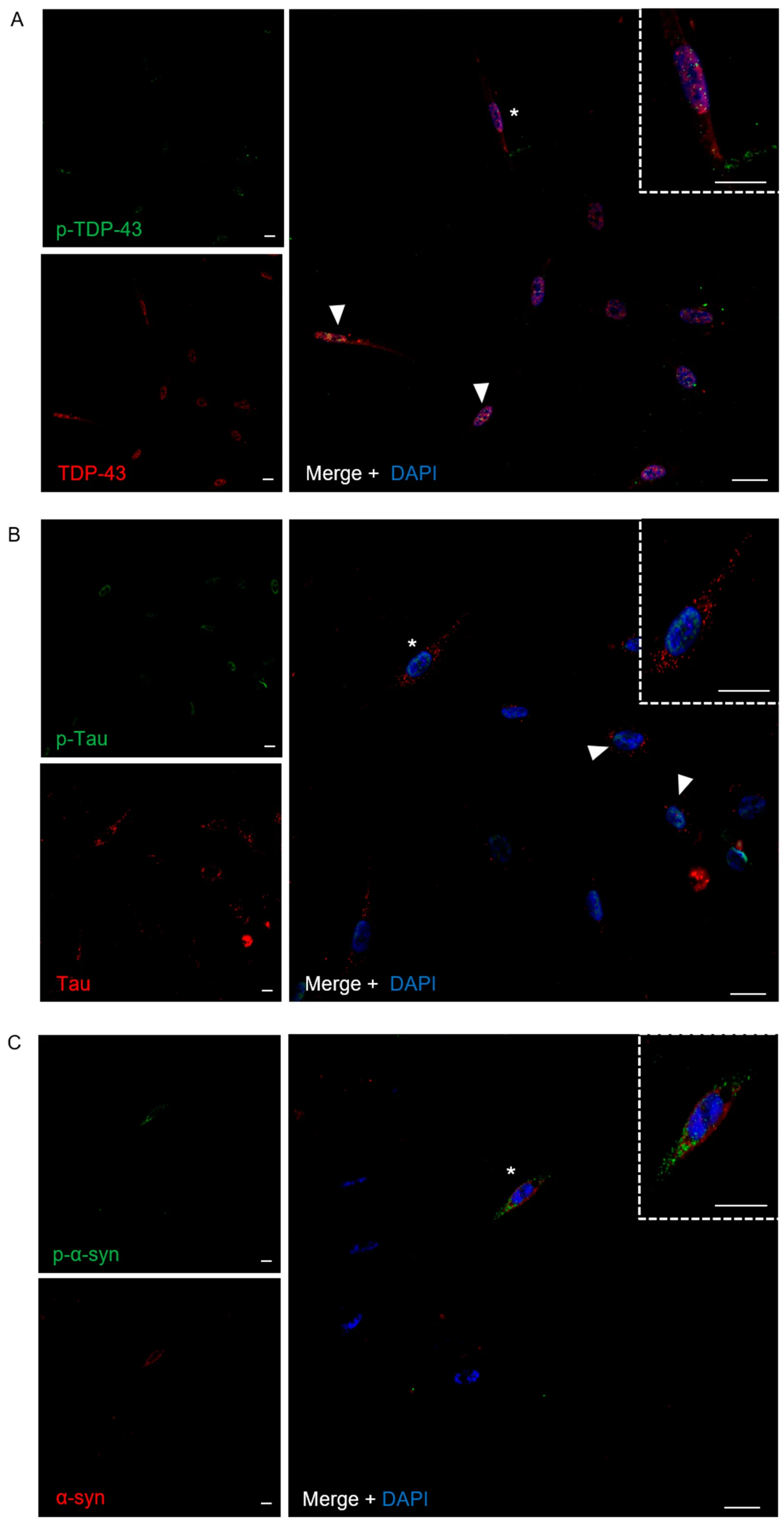
Representative immunofluorescence images of olfactory epithelium cells from healthy individuals at approximately T1 of culture, showing staining of (**A**) pTDP-43 and TDP-43, (**B**) p-Tau and Tau, and (**C**) p-α-syn and α-syn. Arrows indicate positive staining. Insets show magnified views of the indicated regions (*). Nuclei were counterstained with DAPI (blue). Scale bars = 20 µm and 5 µm (inset). Images are from one healthy individual out of 22 analyzed. For each individual, 2–3 slides were stained, and the same staining pattern was observed across slides and subjects.

We characterized the expression and cellular localization of neurodegeneration-associated proteins in hON from healthy individuals (HIs). TDP-43 was detected in the cytoplasm and nuclei; conversely, p-TDP-43 was observed in the cytoplasm (Figure 8A). Tau displayed a cytoplasmic distribution, whereas p-Tau signal was confined to the nucleus. α-syn and p-α-syn showed a predominantly cytoplasmic signal (Figure 8B,C).

**Figure 9:**
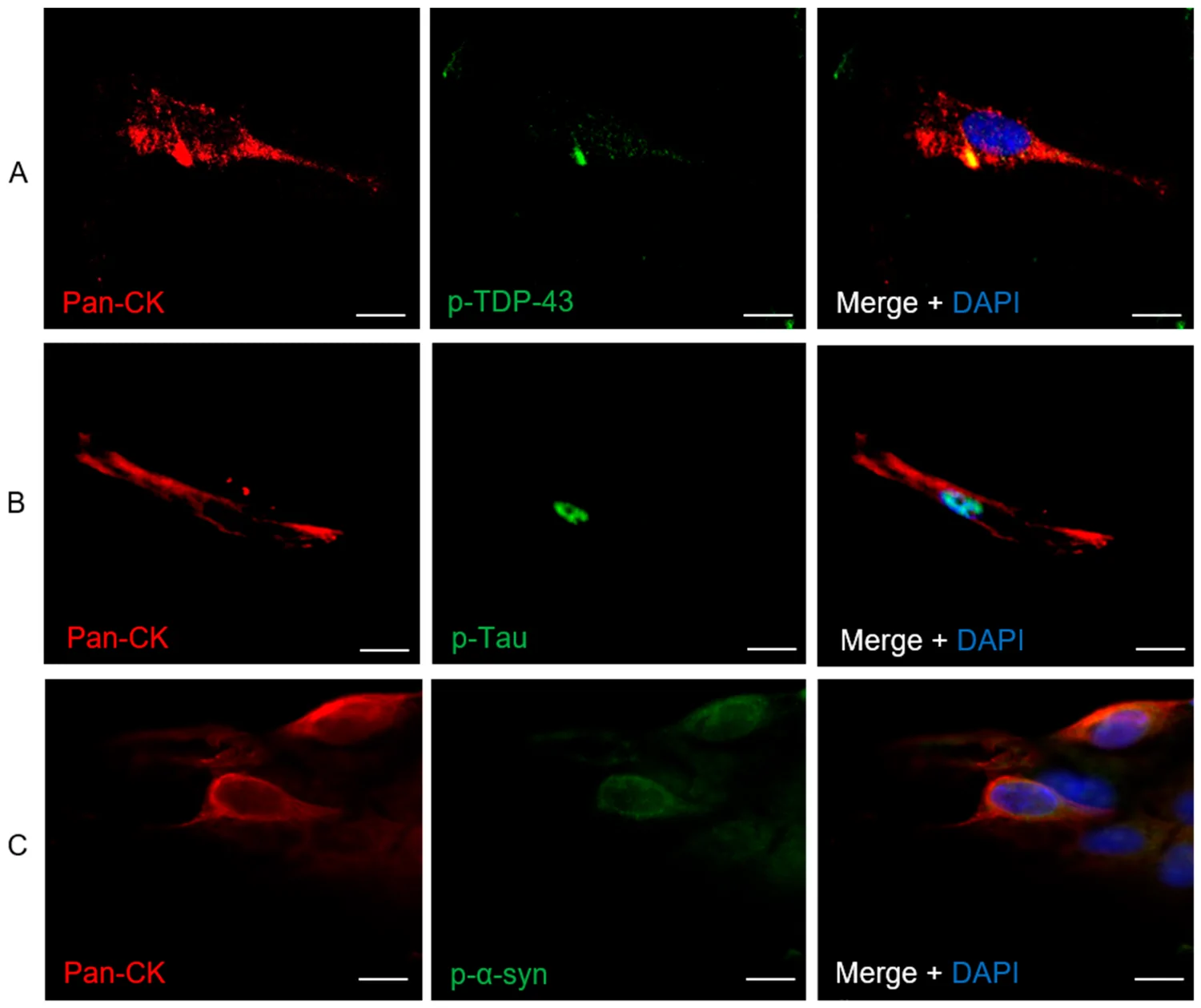
Representative immunofluorescence staining in sustentacular cells Pan-CK^+^ at approximately T1of cells derived from healthy individuals for (**A**) p-TDP-43, (**B**) p-Tau, and (**C**) p-α-syn (Scale bar = 10 μm). Nuclei were counterstained with DAPI (blue). Images are from one healthy individual out of 22 analyzed. For each individual, 2–3 slides were stained, and the same staining pattern was observed across slides and subjects.

**Figure 10:**
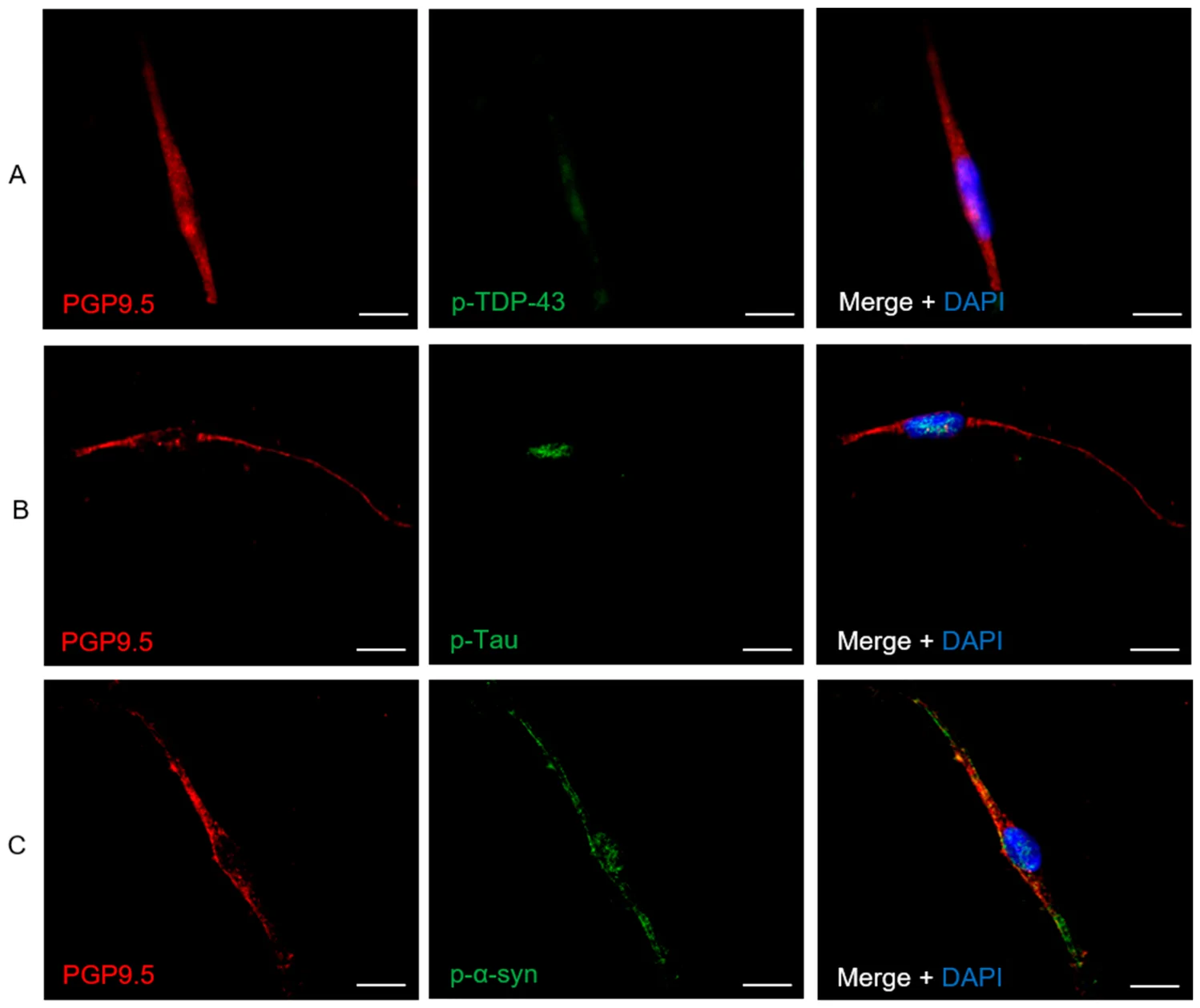
Representative immunofluorescence staining in neuronal cells PGP9.5^+^ at approximately T1 of cells derived from healthy individuals for (**A**) p-TDP-43, (**B**) p-Tau, and (**C**) p-α-syn (Scale bar = 10 μm). Nuclei were counterstained with DAPI (blue). Images are from one healthy individual out of 22 analyzed. For each individual, 2–3 slides were stained, and the same staining pattern was consistently observed in all analyzed samples.

To verify that protein localization was preserved under culture conditions, we next analyzed the cellular distribution of the same proteins and their phosphorylated isoforms in cultured hON. IF staining revealed cytoplasmic localization of p-TDP-43 in Pan-CK^+^ cells, confirming the physiological localization of the protein (Figure 9A). Moreover, p-Tau protein was also detected in the nucleus of Pan-CK^+^ cells (Figure 9B). Regarding p-α-syn, we observed a faint cytoplasmic signal (Figure 9C). In parallel, the presence of neurodegeneration-related proteins was revealed in PGP9.5^+^ neuronal cells. IF staining detected nuclear localization of both p-TDP-43 and p-Tau (Figure 10A,B). Meanwhile, p-α-syn showed a widespread cytoplasmic and nuclear distribution (Figure 10C).

### 3.7. hON Showed a Phenotypic Transition Towards Fibroblast-like Cells at Long-Term Culture

**Figure 11:**
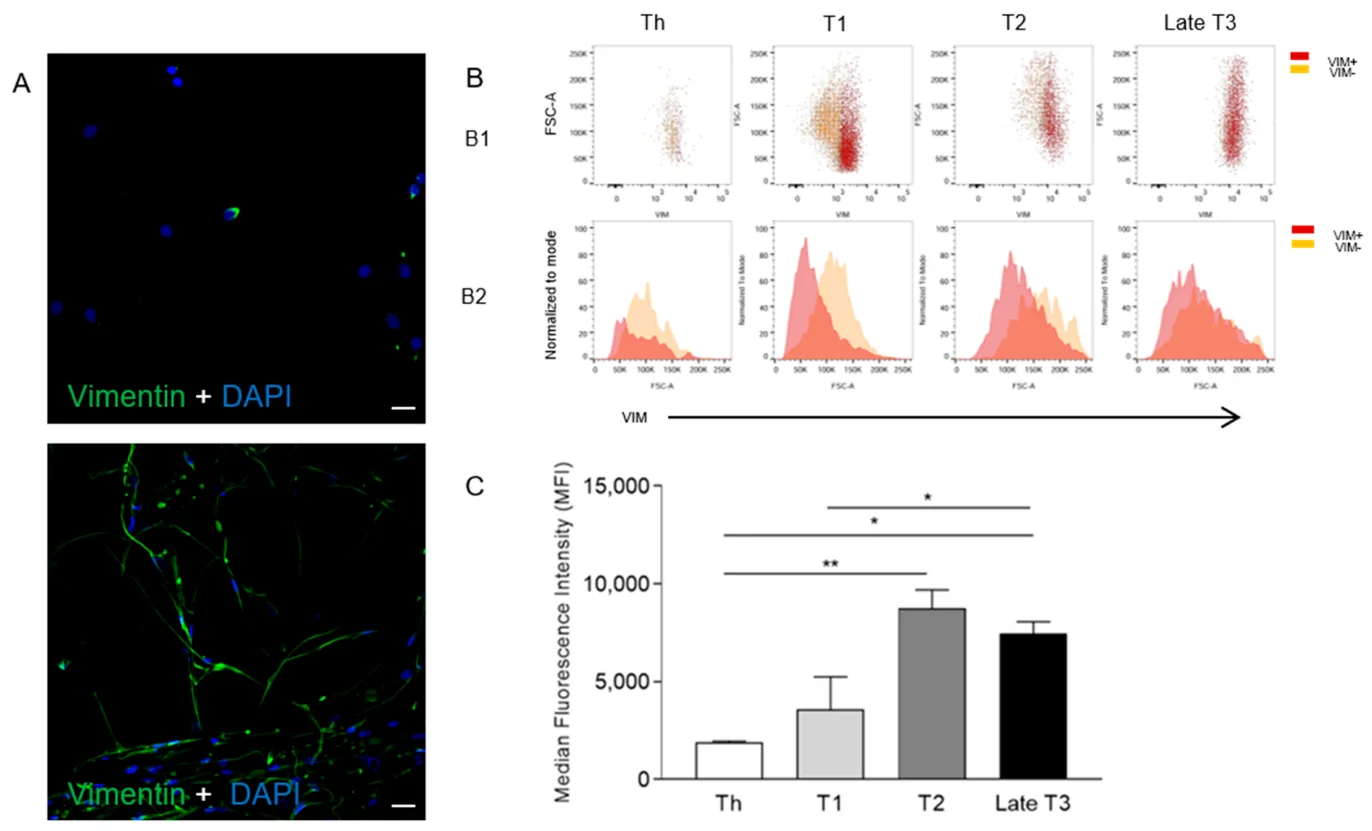
(**A**) Representative images of ON cells stained with vimentin antibody, respectively at the time of harvesting (upper panel) and Late T3 (lower panel) (Scale bar = 20 µm). Images are from one healthy individual out of 22 analyzed. For each individual, 2–3 slides were stained, and the same staining pattern was consistently observed in all analyzed samples. (**B**) Representative overlay dot plots showing the forward scatter parameter (FSC-A) of VIM^+^ (red dots in B1 and histogram in B2) and VIM^−^ (yellow dots in B1 and histogram in B2). (**C**) Median fluorescence intensity (MFI) of the vimentin marker of hON at different time points. Analysis was performed using a one-way ANOVA. Data are shown as mean ± SEM of five healthy individuals (*n* = 5) (Th vs. T2 ** *p* < 0.05, Th vs. Late T3 * *p* < 0.05, and T1 vs. Late T3 * *p* < 0.05).

After 21 days of culture (Late T3), we observed a morphological change in the shape of hON cells. Cells exhibited a transition towards a fibroblast-like morphology, becoming elongated and spindle-shaped with multiple extended processes, and were positive for vimentin antibody (Figure 11A). The morphological change was confirmed by the FSC-A analysis of vimentin^+^ cells over time (Figure 11B). In particular, flow cytometry analysis of FSC-A across sequential time points demonstrated a clear temporal shift in cell size. In addition, the transition of cells toward a fibroblast-like phenotype was further corroborated by vimentin MFI values at distinct time points. A progressive increase in MFI was detected from Th to T2, followed by a slight decrease at T3 (Th vs. T2 *p* = 0.0057, Th vs. Late T3 *p* = 0.0188 and T1 vs. Late T3 *p* = 0.0273) (Figure 11C).

## 4. Discussion

Human olfactory neuroepithelium cell culture (hON) represents a valuable source of neural material from living subjects, capable of reproducing physiological and pathological features of in vivo cells, making it a promising in vitro model for neurodegenerative diseases (NDs).

In our previous pioneering work, the nasal swab (NS) has been shown to be a reliable and non-invasive method to access neural cells in vivo [10]. Following our previous study, we here established a protocol for isolating and co-culturing distinct hON cellular populations using NS. Unlike more invasive biopsy-based approaches, which require tissue excision and a subsequent dissociation procedure [14,26], NS enables multiple and non-invasive sampling and is particularly well-suited for longitudinal monitoring of dynamic cellular and molecular changes [18]. To date, many paradigms have been used to cultivate and characterize olfactory neuroepithelium (ON) cells, highlighting significant inconsistencies in culture conditions and marker expression [27]. In our samples, ON complexity was observed: at the time of harvesting, a Pan-cytokeratin (Pan-CK) antibody was used to detect sustentacular cells (SCs) expressing cytokeratins, and βIV-tubulin^+^-ciliated respiratory cells were detected, with variability related to the operator and sampling area [14]. In addition, both mature and immature neurons were detected by Protein gene product (PGP9.5) antibody with the extension of neurite positivity, further supported by GAP-43 and OMP co-staining, which confirmed the presence of neurons at different maturation stages within the same culture. The expression of putative neuronal markers in hON cells is highly variable across studies and depends on culture conditions and the degree of neuronal differentiation.

Markers such as olfactory marker protein (OMP) or βIII-tubulin have been found to be expressed by multiple cell types, and βIII-tubulin in particular has been reported at disproportionately high levels in some studies, raising concerns about its specificity [10,28]. In this context, PGP9.5 proved more reliable in identifying cells with neuronal-like morphology.

In our protocol, primary hON cultures were maintained without neurotrophic factors or highly supplemented media to preserve naïve conditions, with B27 added solely to support neuronal viability rather than induce differentiation [29,30,31]. Analyzing the transition from harvesting to initial culture, we observed the expansion of S and G2/M phase populations, alongside the increase in cell viability, suggesting that the culture supports active, physiological cell proliferation. In particular, Pan-CK^+^ cells, highly represented at harvest, declined progressively over time, while PGP9.5^+^ neuronal cells remained stable for up to 21 days, defining a practical experimental window of this population with high viability and phenotypic stability. After this period, cultures underwent a phenotypic transition toward fibroblast-like cells [32,33]. Further investigations into the differentiation status and stemness potential of hON cells will clarify the underlying biological dynamics. As part of the characterization of ON cells derived from NS samples from healthy individuals (HIs), we assessed the expression of TAR-DNA binding protein 43 (TDP-43), α-synuclein (α-syn), and Tau, along with their phosphorylated counterparts. Phosphorylation targets only a fraction of the total protein pool; thus, complete overlap was not expected. The observed spatial correspondence nonetheless confirms the specificity of the phospho-antibodies. Since aberrant phosphorylation of these three proteins is considered a defining hallmark of neurodegenerative pathology [34,35,36,37], we examined their cellular distribution across sustentacular and neuronal cell populations, both at the time of harvesting and in culture, to assess whether the observed localization was preserved over time. Notably, p-TDP-43 exhibited cytoplasmic localization at the time of collection in both cell types. While phosphorylation at S409/410 is widely regarded as a pathological modification promoting cytoplasmic mislocalization in amyotrophic lateral sclerosis and frontotemporal dementia [38,39], the weak cytoplasmic immunoreactivity observed in healthy subjects may reflect basal phosphorylation associated with physiological turnover rather than overt pathological accumulation. Upon culture, p-TDP-43 redistributed to the nucleus exclusively in neuronal cells, consistent with physiological localization. This may reflect changes in intracellular trafficking and protein turnover through mechanisms such as importin α/β mediated transport [38] and GDE2-dependent regulation of nuclear pore integrity [39], which are absent in SCs. These findings highlight the complexity of interpreting p-TDP-43 localization in hON samples and underscore the need for comparative studies under pathological conditions. The nuclear localization of p-Tau observed in olfactory sensory neurons is consistent with evidence supporting a physiological role for nuclear Tau in neuronal cells. Tau has been detected in the nuclei of both neuronal and non-neuronal cells, where it contributes to DNA protection [40]. Furthermore, the phosphorylation at specific residues appears selectively enriched in the nuclear compartment compared to the cytosol, a signature distinct from the pathological hyperphosphorylation characteristic of tauopathies [41]. Notably, this nuclear p-Tau distribution was maintained both at sampling and in culture, suggesting a stable, cell-intrinsic pattern rather than a culture-induced artifact. α-syn is canonically described as a presynaptic neuronal protein; however, glial cells have been shown to express α-syn [42], suggesting that low-level expression in non-neuronal cells is possible, as observed in Pan-CK^+^ cells in our study. The p-α-syn accumulation observed across cytoplasmic, nuclear, and process compartments in PGP9.5^+^ cells may similarly reflect physiological synaptic activity. α-syn pS129 has been shown to function as an activity-dependent regulator that accumulates reversibly at presynaptic boutons upon stimulation [43] and appears physiologically present in olfactory structures of healthy subjects across multiple species, where it associates with presynaptic vesicle trafficking proteins [44].

The absence of seeding activity, confirmed by SAA analysis for TDP-43, Tau, and α-syn, further supports the interpretation that the phosphorylated proteins identified in HIs correspond to physiological protein conformation rather than early pathological aggregates.

To the best of our knowledge, this study provides proof of concept that a human cell culture can be established from ON and maintain stable key features in vitro, specifically in terms of cell population composition preserving neuronal and supporting markers and the subcellular localization of neurodegeneration-associated proteins. Consequently, T1 could represent the optimal operational window for high-resolution transcriptomics analyses, protein misfolding and trafficking assays, and short pharmacological testing. However, its finite 2D lifespan precludes chronic long-term studies.

The inherent inter-individual variability in initial cell yield, confluence, and growth rates represents a technical limitation typical of primary human cultures derived from living donors. Nevertheless, while quantitative cell recovery may vary between individuals, cellular morphology, marker expression, and protein localization in PGP9.5^+^ and PanCK^+^ cells remain consistent across donors during the optimal culture window.

While the relatively small sample size is a limitation, this work provides evidence for the feasibility of hON culture from NS. The present study was designed to establish and characterize hON cultures derived from healthy individuals; therefore, ON samples from neurodegenerative patients were included exclusively as SAA controls and were not investigated for protein localization or culture-related changes. Comparative analyses with patient-derived cultures will be necessary to discriminate physiological phenomena from pathological alterations; future studies in larger cohorts will confirm and extend these findings. Overall, our findings support the feasibility of establishing and maintaining hON cultures from nasal swab-derived olfactory neuroepithelium and suggest their potential utility as a non-invasive platform for future investigations of neurodegenerative disease-related mechanisms, including protein misfolding and alterations in protein localization and phosphorylation.

## Figures and Tables

**Table 1:** Primary and secondary antibodies used for IF and FC.

**Primary** **Antibody**	**Host**	**Company**	**Catalogue Code**	**IF Dilution**	**FC Dilution**
anti-PGP9.5	Mouse	GeneTex, Irvine, CA, USA	GTX634797	1:200	1:200
anti-βIV-tubulin	Mouse	Sigma-Aldrich, St. Louis, MO, USA	T7941	1:300	1:200
anti-βIII-tubulin	Rabbit	Sigma-Aldrich, St. Louis, MO, USA	T2200	1:250	1:300
anti-Pan-CK	Mouse	Invitrogen, Carlsbad, CA, USA	MA5-13156	1:300	1:300
anti-CK5	Rabbit	Abcam, Cambridge, UK	ab53121	1:300	n.a
anti-CK8	Rat	Merck Life Science, Milano, Italy	MAB329	1:300	n.a
Anti-CK18	Mouse	Invitrogen, Carlsbad, CA, USA	MA5-12104	1:300	n.a
anti-GAP-43	Rabbit	Merck Life Science, Milano, Italy	AB5220	1:100	n.a
anti-OMP	Goat	Wako, Osaka, Japan	544-10001	1:80	n.a
anti-p-TDP-43 (S409/410)	Rat	Biolegend, San Diego, CA, USA	829901	1:800	n.a
anti-TDP-43 (N-terminal)	Rabbit	Sigma-Aldrich, St. Louis, MO, USA	SAB4200006	1:200	n.a
anti-p-α-synuclein (S129)	Rabbit	Invitrogen, Carlsbad, CA, USA	PA5-77815	1:500	n.a
anti-α-synuclein	Mouse	Biolegend, San Diego, CA, USA	834301	1:200	n.a
anti-p-Tau (S199)	Rabbit	Invitrogen, Carlsbad, CA, USA	44734G	1:400	n.a
anti-Tau	Mouse	Invitrogen, Carlsbad, CA, USA	MN1000	1:400	n.a
anti-Vimentin	Chicken	Sigma-Aldrich, St. Louis, MO, USA	AB5733	1:400	1:400
**Secondary** **Antibodies**	**Host**	**Company**	**Catalogue Code**	**IF Dilution**	**FC Dilution**
anti-goat	Donkey	Invitrogen, Carlsbad, CA, USA	A-11055	1:1000	n.a
anti-mouse	Donkey	Invitrogen, Carlsbad, CA, USA	A-31571	1:1000	1:2500
anti-mouse	Goat	Invitrogen, Carlsbad, CA, USA	A-11001	1:1000	1:2000
anti-rabbit	Goat	Invitrogen, Carlsbad, CA, USA	A-11008	n.a	1:2000
anti-rabbit	Goat	Invitrogen, Carlsbad, CA, USA	A-11012	1:1000	n.a
anti-rat	Goat	Invitrogen, Carlsbad, CA, USA	A-11006	1:1000	n.a
anti-chicken	Goat	Invitrogen, Carlsbad, CA, USA	A-11039	1:1000	1:2500

n.a: not applicable.

**Table 2:** Expression of Pan-CK and PGP9.5 in ON cells from HIs at Th and during culture.

		Th	T1	T2	T3
Pan-CK	Positive cells (%)	60.4 ± 5.5	30.4 ± 7.7	10.6 ± 6.9	2 ± 0.75
	MFI ± SEM	968.2 ± 212.1	217.8 ± 58.9	56.4 ± 18.5	84 ± 27.4
PGP9.5	Positive cells (%)	31.2 ± 4.2	45.8 ± 7.3	38.6 ± 7.2	32.5 ± 6.1
	MFI ± SEM	445.6 ± 46.4	466.2 ± 104.3	772 ± 150.7	725 ± 169.4

## Data Availability

The original contributions presented in this study are included in the article/Supplementary Material. Further inquiries can be directed to the corresponding authors.

## Supplementary Materials

The following supporting information can be downloaded at: https://www.mdpi.com/article/10.3390/cells15191824/s1, Figure S1: Representative images of ON cells at the time of harvesting, expressing CK8 (A), CK18 (B), and CK5 (C). Scale bar = 20 µm. Nuclei were counterstained with DAPI. Images are from one healthy individual out of 22 analysed. For each individual, 2–3 slides were stained and the same staining pattern was consistently observed in all analysed samples. Figure S2: Representative images of ON cells from NS after sampling. Neuronal expression of βIII-tubulin (A) and PGP9.5 (B). Scale bar = 20 µm. Nuclei were counterstained with DAPI. Images are from one healthy individual out of 22 analysed. For each individual, 2–3 slides were stained and the same staining pattern was consistently observed in all analysed samples. (C) Median fluorescence intensity (MFI) of βIII-tubulin and PGP9.5 marker of ON cells at harvest time. Analysis was performed using a paired *t*-test, and data are shown as mean ± SEM (standard error of the mean) of five healthy individuals (*n* = 5). * *p* < 0.02. Figure S3: Representative brightfield light microscopy images illustrating cell morphology, attachment, and confluence progression of human olfactory neuroepithelial (hON) cells in culture from harvesting (Th) across culture time points. Images are from one healthy individual out of 22 analysed. Scale bar = 200 μm. Figure S4: Representative immunofluorescence images of PGP9.5^+^ cells in hON cultures at T1, co-stained with Phalloidin. Double staining for Phalloidin (green, F-actin) and PGP9.5 (red) in a representative cell, showing the full cellular extent outlined by F-actin. Scale bar = 20 µm. Nuclei were counterstained with DAPI. Figure S5: Representative immunofluorescence images of PGP9.5^+^ and OMP^+^ cells in hON cultures at T1, co-stained with GAP-43. (A) Double staining for GAP-43 (green) and PGP9.5 (red); arrowheads indicate GAP-43 immunoreactivity restricted to the proximal segment of the PGP9.5^+^ process. (B) Double staining for OMP (green) and GAP-43 (red) showing the different maturation stages of neurons. Scale bar = 20 µm. Nuclei were counterstained with DAPI.

## Abbreviations

BCs	basal cells
FMO	fluorescence minus one control
HIs	healthy individuals
hON	human ON cell culture
IF	immunofluorescence
iPSCs	induced pluripotent stem cells
NSs	nasal swabs
NDs	neurodegenerative diseases
OM	olfactory mucosa
ON	olfactory neuroepithelium
OSNs	olfactory sensory neurons
OS	olfactory system
Pan-CK	Pan-cytokeratin
PFA	paraformaldehyde
PGP9.5	Protein gene product 9.5
RT	room temperature
SAA	Seeding Amplification Assay
SCs	sustentacular cells
TDP-43	TAR-DNA binding protein 43
Th	time of harvesting
α-syn	α-synuclein
